# How accurate is the reporting of stroke in hospital discharge data? A pilot validation study using a population-based stroke registry as control

**DOI:** 10.1007/s00415-012-6686-0

**Published:** 2012-10-18

**Authors:** Corine Aboa-Eboulé, Dominique Mengue, Eric Benzenine, Marc Hommel, Maurice Giroud, Yannick Béjot, Catherine Quantin

**Affiliations:** 1 Stroke Registry of Dijon, EA 4184, University Hospital and Faculty of Medicine of Dijon, STIC-Santé, University of Burgundy, Dijon, France; 2 Département d’Informatique Médicale, University Hospital of Dijon, Dijon, France; 3 INSERM U666, University of Burgundy, Dijon, France; 4 CIC, CHU de Grenoble, La Tronche, France; 5 Service de Neurologie, CHU Dijon, BP 77908, 21079 Dijon CEDEX, France

**Keywords:** Stroke, Registry, Hospital discharge data, Validation, Administrative data

## Abstract

Population-based stroke registries can provide valid stroke incidence because they ensure exhaustiveness of case ascertainment. However, their results are difficult to extrapolate because they cover a small population. The French Hospital Discharge Database (FHDDB), which routinely collects administrative data, could be a useful tool for providing data on the nationwide burden of stroke. The aim of our pilot study was to assess the validity of stroke diagnosis reported in the FHDDB. All records of patients with a diagnosis of stroke between 2004 and 2008 were retrieved from the FHDDB of Dijon Teaching Hospital. The Dijon Stroke Registry was considered as the gold standard. The sensitivity, positive predictive value (PPV), and weighted kappa were calculated. The Dijon Stroke Registry identified 811 patients with a stroke, among whom 186 were missed by the FHDDB and thus considered false-negatives. The FHDDB identified 903 patients discharged following a stroke including 625 true-positives confirmed by the registry and 278 false-positives. The overall sensitivity and PPV of the FHDDB for the diagnosis of stroke were, respectively, 77.1 % (95 % CI 74.2–80) and 69.2 % (95 % CI 66.1–72.2). For cardioembolic and lacunar strokes, the FHDDB yielded higher PPVs (respectively 86.7 and 84.6 %; *p* < 0.0001) than those of other stroke subtypes. The PPV but not sensitivity significantly increased over the years (*p* < 0.0001). Agreement with the stroke registry was moderate (kappa 52.8; 95 % CI 46.8–58.9). The FHDDB-based stroke diagnosis showed moderate validity compared with the Dijon Stroke Registry as the gold standard. However, its accuracy (PPV) increased with time and was higher for some stroke subtypes.

## Introduction

The organization of care networks is essential to limit the adverse consequences of stroke [1]. Reliable estimates of the stroke burden at a national level is therefore required to establish efficient health policy regarding needs in terms of health services and primary and secondary vascular prevention. The gold standard for the assessment of stroke incidence is population-based registries, which ensure the exhaustiveness of case ascertainment by identifying fatal and non-fatal strokes [2]. However, the data obtained from population-based registries cannot reflect disparities across the country since they cover relatively small populations.

In France, only one stroke registry, in the city of Dijon, has been maintained since 1985. More recently, the French Hospital Discharge Database (FHDDB) was developed to routinely collect administrative data in acute-care hospitals. Even though it was originally created to determine the financial requirements of hospitals throughout France, it could be a useful tool to evaluate the nationwide burden of stroke [3] since the approach has been used in other countries [4–7]. However, the FHDDB has not yet been validated for stroke diagnosis.

The first aim of this study was to assess the validity of the FHDDB for the diagnosis of stroke from 2004 to 2008. To achieve this goal, we used the Dijon Stroke Registry as the gold standard. The second aim was to identify potential sources of errors, false-positives, and false-negatives, in medical records.

## Materials and methods

### Study setting

#### FHDDB

The FHDDB was adapted from the American Diagnosis-related Group (DRG) in 1991 [8]. This system compares resource utilization across groups of patients with the same principal diagnosis and can be used to provide an estimation of cost per DRG. The objectives of the FHDDB, implemented in 1998, were to evaluate the activity of public hospitals and thereby to establish their financial requirements. Since 2004, the FHDDB has become exhaustive for hospital inpatient claims because the financial resources of public and private hospitals depend on a DRG prospective payment system. The DRG scheme relies on anonymous discharge abstracts, which include administrative and medical data recorded in the FHDDB for each stay. In France, according to health policy, attending physicians are responsible for the coding of hospital discharge abstracts for their patients. In practice, however, several situations are possible: most of the time, a junior doctor codes the abstract; sometimes, the medical secretary prepares the abstract, which is then validated by a senior physician or physicians themselves fill in the abstract directly. Diagnoses are coded using the International Classification of Diseases, 10th revision (ICD-10) either as primary (condition associated with the greatest use of resources), or secondary (related significant associated diagnosis). Procedures are coded using the French Common Classification of Medical procedures (CCAM). The discharge abstract is then included in one DRG according to classification variables such as diagnoses, procedures, and demographic characteristics. Every month, the hospitals transmit all their administrative discharge abstracts to the national center (FHDDB) via a dedicated site.

#### Dijon Stroke Registry

The Dijon Stroke Registry has prospectively collected all stroke cases occurring in the city of Dijon since 1985 (2008 census: 155,125 inhabitants). Briefly, the case ascertainment procedure relies on multiple overlapping sources of information to identify fatal and non-fatal strokes patients. Data are obtained from: (1) the emergency rooms, and clinical and radiological departments of Dijon Academic Hospital and three private hospitals, (2) the patient’s home or nursing homes, with diagnosis assessed by the general practitioners helped by neurologists from outpatients clinics, (3) the records of radiological and Doppler ultrasound centers, and (4) the death certificates obtained from the local Social Security Bureau. All diagnoses of stroke are validated within the registry. Further details on registry organization have been provided elsewhere [9].

### Study population

#### FHDDB of Dijon Teaching Hospital

**Fig. 1:**
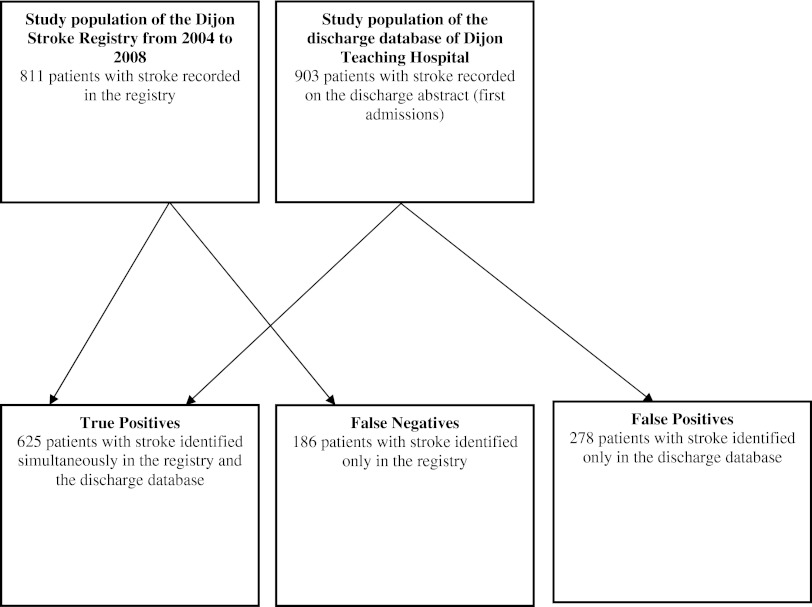
Flow diagram

Abstracts of patients diagnosed with stroke from January 1, 2004 to December 31, 2008, and who were residents of the city of Dijon were retrospectively extracted from the FHDDB of Dijon Teaching Hospital. An algorithm was applied to select primary diagnoses with one of the following ICD-10 codes: I61, I63, I64, and G46 [10]. Exclusion criteria were ICD-10 codes for transient ischemic attacks (G45) and subarachnoid hemorrhage (I60). All hospitalizations with a diagnosis of stroke were considered. Strokes were then classified in six subtypes: lacunar infarct, ischemic stroke from cardiac embolism, large-artery atherosclerosis, ischemic strokes from other etiologies, intracerebral hemorrhage, and strokes from unknown etiologies. The final study population included 903 stroke cases (Fig. 1).

#### Dijon Stroke Registry

A total of 811 stroke cases, hospitalized in Dijon Teaching Hospital from January 1, 2004 to December 31, 2008, were retrospectively extracted from the registry (Fig. 1). First-ever and recurrent strokes were considered. Stroke was defined according to the World Health Organization recommendations and the International Classification of Disease [9]. The stroke subtype was diagnosed on a clinical examination together with cerebral imaging, two-dimensional echocardiography, carotid and vertebral ultrasonography, and standard blood and urine tests. We grouped strokes in five subtypes as follows: lacunar infarct, ischemic stroke from cardiac embolism, large-artery atherosclerosis, ischemic strokes from other etiologies, and spontaneous intracerebral hemorrhage. Transient ischemic attacks and subarachnoid hemorrhage were excluded.

### Anonymous linkage of FHDDB and registry database

Anonymous identifiers were created to link the FHDDB and the registry database to respect French legislation [4, 11–13]. These identifiers were nominative data such as the last name, first name, and date of birth, which were rendered anonymous using irreversible hash coding in each database with our ANONYMAT software [12].

Records from the FHDDB were anonymously matched with those of the registry database using probabilistic record linkage [12] taking into account identity entry errors. Records were matched by anonymized last names, first names, and dates of birth.

### Data analyses

The baseline characteristics of patients selected in the FHDDB and the registry were compared.

The validity (ability to correctly diagnose stroke cases) and accuracy (the extent to which the stroke coding reflects the underlying patient’s stroke pathology) of the FHDDB-based diagnosis of stroke were assessed using sensitivity and PPV. False-positives (FP) were defined as cases recorded in administrative (FHDDB) data, but not in the Dijon Stroke Registry. In the same way, cases recorded in the Dijon Stroke Registry but not in the FHDDB were considered false-negatives (FN). Ninety-five percent confidence intervals (95 % CI) were calculated for overall sensitivity and PPV. We stratified characteristics according to patient status (TP, FN, and FP) to examine whether indicators varied across strata. True-positives (TP), simultaneously diagnosed by the FHDDB and the registry, FN, and FP were determined. The sources of errors in the FHDDB-based stroke diagnosis were analyzed by reviewing the available complete medical records of false-positives and false-negatives. The weighted kappa statistic, which is appropriate for variables with more than two categories, was used to measure the agreement between stroke subtypes recorded in the FHDDB and in the registry. This kappa analysis was restricted to 599 TP since 26 patients with strokes of unknown etiology in the FHDDB were excluded. Differences in kappa coefficient per year were examined. Respective kappa values of 0.41–0.60, 0.61–0.80, and 0.81–1.00 were considered moderate, or good, or very good [14]. The Chi-square test was used for heterogeneity. A two-sided significance was set at *p* < 0.05. SAS 9.2 (SAS Institute, Inc.) was used for statistical analyses.

## Results

The 903 patients selected from the FHDDB and the 811 patients from the registry were compared according to baseline characteristics. They were similar for age (mean age 75.41 ± 15.53 and 75.38 ± 14.79, respectively) and gender. However, they differed significantly according to the stroke subtype and year of admission. The proportion of strokes from: (1) intracerebral hemorrhage was 14.2 % for the FHDDB versus 13.2 % for the registry, (2) cardiac embolism was 15.0 % for the FHDDB versus 23.7 % for the registry; (3) large-artery atherosclerosis was 17.3 % for the FHDDB versus 31.3 % for the registry; (4) lacunar infarct was 8.6 % for the FHDDB versus 20.2 % for the registry; (5) ischemic strokes related to other etiologies was 37.1 % for the FHDDB versus 11.6 % for the registry; from strokes related to unknown etiologies was 7.9 % for the FHDDB and 0 % for the registry (*p* < 0.0001).

The annual rates of stroke were: (1) in 2004, 22.0 % for the FHDDB versus 15.7 % for the registry; in 2005, 19.5 % for the FHDDB versus 18.1 % for the registry; in 2006, 17.1 % for the FHDDB versus 20.2 % for the registry; in 2007, 20.8 % for the FHDDB versus 23.7 % for the registry; in 2008, 20.6 % for the FHDDB versus 22.3 % for the registry (*p* = 0.007). A total of 625 patients were TP simultaneously diagnosed by the FHDDB and the registry while 186 were FN and 278 were FP.

The overall sensitivity and PPV of the FHDDB-based stroke diagnosis were 77.1 % (95 % CI 74.2–80 %) and 69.2 % (95 % CI 66.2–72.2 %) as shown in Table 1. Sensitivity and PPV were heterogeneous across age strata with higher values for patients aged 70 and more than for patients aged under 70 (*p* for heterogeneity = 0.013 and 0.005). Neither sensitivity nor PPV differed according to gender, but they did for stroke subtypes. The diagnosis of intracerebral hemorrhage in the FHDDB was associated with the highest sensitivity (87.9 %) despite the lowest PPV (64.8 %) related to a high frequency of FP (16.2 %, over a total of 45 FP and 83 VP in the FHDDB). The results for other subtypes were consistent for both indicators with higher values for cardioembolic stroke and lower values for large-artery atherosclerosis and ischemic strokes from other etiologies. The diagnosis of lacunar infarct was associated with a high PPV (84.6 %) but moderate sensitivity (73.2 %). There was a gradual rise in PPV with time from 54.3 % in 2004 to 81.2 % in 2008 (*p* for heterogeneity < 0.0001), which was not observed for sensitivity (Table 1).

**Table 1:** Sensitivity and positive predictive value of the FHDDB using the Dijon Stroke Registry as the gold standard

	True-positives^a^ *n* (%)	False-positives^b^ *n* (%)	False-negatives^b^ *n* (%)	Sensitivity (%)	Positive predictive value (%)
Total	625 (100.0)	278 (100.0)	186 (100.0)	77.1	69.2
Age					
<70	142 (22.7)	88 (31.7)	59 (31.7)	70.6	61.9
≥70	483 (77.3)	190 (68.3)	127 (68.3)	79.2	71.7
*p* for heterogeneity^c^				0.013	0.005
Gender					
Female	357 (57.1)	147 (52.9)	95 (51.1)	79.0	70.8
Male	268 (42.9)	131 (47.1)	91 (48.9)	74.7	67.2
*p* for heterogeneity^c^				0.145	0.236
Stroke subtypes					
Intracerebral hemorrhage	94 (15.0)	45 (16.2)	13 (7.0)	87.9	64.8
Ischemic stroke from cardiac embolism	160 (25.6)	18 (6.5)	32 (17.2)	83.3	86.7
Large-artery atherosclerosis	188 (30.1)	44 (15.8)	66 (35.5)	74.0	71.8
Lacunar infarct	120 (19.2)	12 (4.3)	44 (23.7)	73.2	84.6
Ischemic strokes from other etiologies	63 (10.1)	114 (41.0)	31 (16.7)	67.0	66.0
Strokes from unknown etiologies	0 (0.0)	45 (16.2)	0 (0.0)	–	36.6
*p* for heterogeneity^c^				0.0007	<0.0001
Year of admission					
2004	102 (16.3)	91 (32.7)	25 (13.4)	80.3	54.3
2005	111 (17.8)	66 (23.7)	36 (19.4)	75.5	62.5
2006	116 (18.6)	39 (14.0)	48 (25.8)	70.7	74.7
2007	146 (23.4)	47 (16.9)	46 (24.7)	76.0	75.0
2008	150 (24.0)	35 (12.6)	31 (16.7)	82.9	81.2
*p* for heterogeneity^c^				0.083	<0.0001

^a^Strokes correctly identified by the FHDDB

^b^Inconsistencies between the FHDDB and the stroke registry

^c^ *p* value for heterogeneity obtained by comparing above differences in sensitivity and positive predictive values using Chi-square test

Table 2 presents the analysis of false-positives of the FHDDB with the registry as the gold standard. The false-positives were patients identified in the FHDDB (with a stroke diagnosis coded in the abstract) but who were not found in the stroke registry. For these patients, we went back to the medical charts stored in the medical departments to understand the reasons for these errors. For example, in 2004: (1) there were 29 patients with a stroke diagnosis coded in the FHDDB but not recorded in the registry and without a stroke diagnosis in the medical chart. Indeed, there was a coding error in the abstract of the FHDDB for these patients. Among them, a stroke diagnosis was coded instead of another diagnosis for 25 patients. For four others, a stroke diagnosis was coded although the patients had in fact presented a transient ischemic attack; (2) For 12 other patients, the stroke diagnosis was mentioned both in the FHDDB and the medical chart, but for six of them, it was in fact a sequel of stroke (prior stroke). For six others, there was an error in the ZIP code of the abstract of the FHDDB because these patients were not residents of the city of Dijon and were thus not recorded in the registry.

**Table 2:** False-positives analysis (FHDDB vs. stroke registry used as a gold standard) from 2004 to 2008

Year of admission	2004 *n* (%)	2005 *n* (%)	2006 *n* (%)	2007 *n* (%)	2008 *n* (%)	Total *n* (%)
No mention of stroke diagnosis in the medical chart						
Incorrect ICD-10 code (*n* = 63)	25 (61.0)	13 (59.0)	5 (50)	12 (60)	8 (66.7)	63 (60)
Stroke coded as transient ischemic attack (*n* = 15)	4 (9.8)	4 (18.2)	3 (30)	3 (15)	1 (8.3)	15 (14.3)
Mention of stroke diagnosis in the medical chart						
Errors in the facility site number (patient hospitalized for stroke elsewhere, *n* = 11)	6 (14.6)	1 (4.6)	1 (10)	2 (10)	1 (8.3)	11 (10.5)
Errors in the patient’s ZIP code (*n* = 16)	6 (14.6)	4 (18.2)	1 (10)	3 (15)	2 (16.7)	16 (15.2)
Total false-positive analysed^a^	41 (100)	22 (100)	10 (100)	20 (100)	12 (100)	105 (100)

^a^Number of false-positives = 105

False-negatives were patients with a stroke diagnosis recorded in the stroke registry (considered the gold standard) but who were not identified in the FHDDB. Like for the FP, we went back to the medical charts of the FHDDB to explore the reasons for the discordances between the stroke registry and the FHDDB. We examined the 169 medical charts that were available (Table 3). On the one hand, FN mostly concerned patients with no mention of stroke as the primary diagnosis in the FHDDB (*n* = 151 out of 169, 89 %). For each year of admission, transient ischemic attacks and related syndromes accounted for roughly one-third of FN (31.4 %). Hemiplegia, tetraplegia, and other paralytic syndromes accounted for about one-quarter (26.6 %). We found that stroke was correctly diagnosed) for the 151 FN during their hospital stay and these patients were actually cared for their stroke. However, there was a coding error in the FHDDB for 90 of them (59.6 %). For the other 61 (40.4 %), the stroke event was coded as the secondary diagnosis in the FHDDB. As a result, they were not selected in the FHDDB when the primary diagnosis algorithm was applied.

**Table 3:** Analysis of false-negatives (FHDDB vs. stroke registry used as the gold standard) from 2004 to 2008

Year of admission	2004 *n* (%)	2005 *n* (%)	2006 *n* (%)	2007 *n* (%)	2008 *n* (%)	Total *n* (%)
No mention of stroke diagnosis in the medical chart (coding errors)						
1. Diagnosis related to nervous system (*n* = 119)						
Transient cerebral ischemic attacks and related syndromes (G45)	8 (38.1)	7 (25)	11 (25.6)	17 (38.6)	10 (30.3)	53 (31.4)
Other non-traumatic intracranial hemorrhage (I62); Sequelae of cerebrovascular disease (I69)	2 (9.5)	0 (0.0)	2 (4.6)	1 (2.3)	6 (18.2)	11 (6.5)
Occlusion and stenosis of precerebral arteries, not resulting in cerebral infarction (I65)	0 (0.0)	0 (0.0)	0 (0.0)	0 (0.0)	2 (6.1)	2 (1.2)
Hemiplegia (G81); paraplegia and tetraplegia (G82); other paralytic syndromes (G83)	0 (0.0)	12 (42.9)	14 (32.6)	18 (40.9)	1 (3.0)	45 (26.6)
Other disturbances of cerebral blood flow: visual disturbances (H53); vascular dementia (F01); vascular syndromes of brain in cerebrovascular diseases (G46)	0 (0.0)	2 (7.1)	3 (7.0)	0 (0.0)	0 (0.0)	5 (2.9)
Epilepsy (G40); status epilepticus (G41)	3 (14.3)	0 (0.0)	0 (0.0)	0 (0.0)	0 (0.0)	3 (1.8)
2. Other diagnoses (*n* = 32)						
Disorders of vestibular function (H81)	0 (0.0)	0 (0.0)	3 (7.0)	1 (2.3)	1 (3.0)	5 (2.9)
Other sepsis (A41); pneumonitis due to solids and liquids (J69)	0 (0.0)	0 (0.0)	1 (2.3)	0 (0.0)	1 (3.0)	2 (1.2)
Symptoms involving the skin (disturbance of skin sensation R20), the nervous system (abnormalities of gait and mobility R26), cognitive function (R41), speech (R47); headache (R51)	2 (9.5)	2 (7.1)	9 (20.9)	2 (4.5)	4 (12.1)	19 (11.2)
Miscellaneous: Polyneuropathy in diseases classified elsewhere (G63); paralytic ileus and intestinal obstruction without hernia (K56,); injury (fracture of femur S72); follow-up examination after treatment for conditions other than malignant neoplasms (Z09); other surgical follow-up care (Z48); Other medical care (Z51)	1 (4.8)	2 (7.1)	0 (0.0)	1 (2.3)	2 (6.1)	6 (3.6)
Mention of stroke diagnosis in the medical chart						
Errors in the patient’s ZIP code (*n* = 18)	5 (23.8)	3 (10.7)	0 (0.0)	4 (9.1)	6 (18.2)	18 (10.7)
Total false-negatives analyzed^a^	21 (100)	28 (100)	43 (100)	44 (100)	33 (100)	169 (100)

^a^Number of false-negatives = 169

For the false-negatives concerning the remaining 18 patients (10.7 %) with a mention of stroke as primary diagnosis in the FHDDB (I63 ICD-10 code in all cases), there was an error in the ZIP code for their place of residence.

Agreement between stroke subtypes reported in the FBDDB and in the stroke registry yielded a moderate weighted kappa statistic (52.8; 95 % CI = 46.8–58.9). There was no significant variation in the kappa statistic between 2004 and 2008 (*p* for homogeneity = 0.420). Values of the kappa statistic were 39.5 % (95 % CI 24.1–54.9 %) in 2004, 50.9 % (35.3–66.5 %) in 2005, 58.8 % (46.6–70.9 %) in 2006, 52.9 % (40.3–65.5 %) in 2007, and 54.4 % (95 % CI 41.6–67.2 %) in 2008.

## Discussion

This study is the first French evaluation of the validity and accuracy of the FHDDB-based diagnosis of stroke using routine administrative data collected over 5 years. Compared with the Dijon Stroke Registry as the gold standard, the sensitivity, PPV, and agreement of the FHDDB were moderate. Our study using a population-based stroke registry with 25 years of experience as the gold standard [9] was an ideal situation for the validation of the FHDDB-based diagnosis of stroke.

Differences in methodology between the FHDDB and the Dijon Stroke Registry may explain the moderate values for sensitivity, PPV, and agreement for the diagnosis of stroke.

In the Dijon Stroke Registry, the ascertainment of stroke cases is exhaustive and continuous through a dedicated professional network, and involves a competent research team for the validation of cases. In contrast, for the FHDDB, the exhaustiveness of case reports has only become a priority since 2004, when discharge abstracts became the basis for hospital funding. In 2005, the FHDDB recorded nearly 130,000 stays for stroke in France, which accounted for almost all strokes cases (95 %) managed in public and private hospitals [15]. These data provided a good estimate of stroke admissions, although the FHDDB is less accurate for the classification of stroke into subtypes, as shown by the moderate kappa statistic in this study. Strokes recorded in the discharge abstracts at the Dijon Stroke Registry are coded by neurologists, whereas those for patients who are hospitalized in departments other than neurology or stroke units, approximately 60 % of strokes, are not [16]. Therefore, the management, diagnosis, and discharge coding is not performed by a stroke specialist. This may lead to coding errors, which were the main sources of FP and FN. To reduce this high proportion of erroneous coding, non-neurologists should receive training so as to improve the accuracy of coding.

Our aim was to know whether the FHDDB-based diagnosis of stroke accurately reflected the underlying stroke pathology. Erroneous coding in the FHDDB is supposed to be low not only for financial incentives but also because the FHDDB may be a source of relevant and useful information in Public Health. The PPV, as a measure of accuracy of the FHDDB-based stroke diagnosis, is useful for clinical research [17]. We showed that the overall PPV improved over the years of the study, indicating that with time, the number of stroke cases identified in the FHDDB should be similar to that recorded in the stroke registry.

In fact, to our knowledge, our pilot study is the first to assess validation indicators for the diagnosis of stroke as recorded in the FHDDB. In France, there have been few validation studies regarding the FHDDB except for cancer [18] and obstetrics [19, 20]. The results of such studies were consistent with those of international studies that used similar types of databases for validation.

Of course, whether our results regarding stroke can be generalized needs to be examined at the national level. We must therefore study the validity of administrative data for the whole country. This is the reason why we had just received grants to implement a nationwide prospective survey from 50 hospitals throughout France.

At the international level, the validation we propose is suitable for any country where administrative data is systematically gathered. Such countries include the USA, Canada, European countries (Belgium, Denmark, Germany, Hungary, Italy, Portugal, Sweden, and the UK), Australia, Japan, New Zealand, and Singapore. The results of our study are consistent with those of previous studies that reported validation indicators for national administrative data based on a DRG system. These studies were American [21, 22], Finnish [23], Italian [24], Canadian [25–27], and European [3]. The first American study was undertaken in Olmsted County (Rochester, Minnesota) to estimate the validity and accuracy of hospital discharge abstracts against one gold standard, the Rochester Stroke Registry, for the years 1970, 1980, 1984, and 1989 [21]. The overall sensitivity of hospital discharge abstract with a primary diagnosis of stroke was 76 % while the PPV was 60 % among 364 patients with incident and recurrent diagnoses of stroke [21]. Another American study in Washington State assessed the validity of administrative hospital discharge data against a review of medical records chosen as the gold standard from 1990 to 1996. With an algorithm based on the primary diagnosis of stroke among 206 patients, the sensitivity and the PPV were 74 and 88 % for ischemic strokes and 85 and 89 % for intracerebral hemorrhage, respectively. The overall kappa for stroke classification was 0.74 (95 % CI 0.64–0.84) [22]. The validation of the Finnish Hospital Discharge Register and cause of death registers against a population-based stroke registry between 1987 and 1998; the FINSTROKE register yielded overall sensitivity of 85 % and PPV of 86 %. As observed in our study, the sensitivity was higher for intracerebral hemorrhage (94 %) than for ischemic strokes (79 %) [23].

More recently, Palmieri and colleagues [24] reported the experience of a cardiovascular registry that has recorded data for a population aged from 35 to 74 years in eight regions of Italy since 1990. In this study, stroke diagnoses according to hospital discharge databases and death certificates were validated against clinical documentation and MONICA diagnostic criteria. The PPV was 35 % for non-fatal strokes in men, and 36 % in women, with several geographical disparities. For fatal strokes, the PPV was 69 % in men and 73 % in women. The algorithm of this study also included secondary diagnoses, which may explain the differences in PPV with regard to our study, particularly for fatal strokes. Moreover, the major limitation of this study was the lack of completeness for stroke ascertainment in some areas.

The Canadian studies by Saponisk et al. [25] and Tu et al. [26] (1994–2004) evaluated the performance of the hospital discharge database using stroke mortality rates. A third Canadian study [27] concerning the Hospital Mortality database evaluated the medical performance of hospitals using mortality rates of ischemic stroke. The European Cardiovascular Incidences Survey Set [3] demonstrated the feasibility of linking hospital discharge data to death registries for stroke follow-up, but the age limits were too restrictive (45–74 years), and the authors did not assess the quality of stroke diagnosis.

Our study has several strengths. We assessed, for the first time, the validity and accuracy of the FDHHB records concerning the diagnosis of stroke in France. Analyses were based on administrative and clinical data. Stroke cases were validated against a population-based registry, which recruited patients from multiple sources, thus limiting selection biases due to incomplete inclusion. The statistical power of the population was sufficient to allow stratified analyses.

Some limitations deserve comment. We chose the primary diagnosis algorithm to select patients with a stroke diagnosis in the FHDDB. However, this algorithm allowed us to have a good PPV even though we may have missed some stroke cases that were incorrectly classified as false-negatives when the stroke event was coded as the secondary diagnosis. The relatively short duration of our study may have hampered temporal trends for metrological indicators. The validation study was performed at a community level and its results cannot be generalized due to regional disparities. The FHDDB included prevalent and incident cases as there was frequent miscoding of the prior stroke event as a primary or secondary diagnosis instead of using the appropriate code for a previous history. Indeed, the hospital discharge database proposed in this study is useful for counting incident cases only because the false-negatives and false-positives tend to have similar frequencies and, thus to cancel each other out. Some individual patients identified through the FHDDB may not necessarily have been diagnosed with stroke, and outpatients diagnosed with stroke may have been missed. Hospital discharge abstracts are unable to accurately identify cases and cannot be used in longitudinal studies. In addition, administrative data do not provide any information about initial stroke severity, as provided, for example, by the National Institute of health Stroke Scale (NIHSS) score, and the degree of functional impairment at discharge using the modified Rankin scale (mRS). Vascular risk factors are coded as secondary diagnoses, which could lead to a lack of exhaustiveness and accuracy. Therefore, stroke registries remain essential to study prognostic factors and to compare stroke care management in different facilities. The classification of stroke subtypes in the FHDDB was not particularly accurate, which yielded a high rate of patients with strokes of unknown etiologies. We found that sensitivity did not increase with time, possibly due to the misclassification of patients as FN because they were discharged, at which time the diagnosis of stroke was recorded, in the year following admission.

In conclusion, the pilot validation study supports the use of routinely collected administrative data for stroke diagnosis. Some issues related to the accuracy of stroke diagnosis and coding were identified, and efforts need to be made to improve the validity and quality of administrative data. The quality of the administrative data recorded in the FHDDB is highly dependent on both the quality of documentation in the medical charts and the experience and expertise of the coder. Several recommendations could be proposed. First, administrative data could be coded in real-time soon after discharge of the patient to avoid delays in coding that could lead to a lack of accuracy and incomplete data. Second, the quality of documentation in medical charts about stroke must be given a high priority in courses for undergraduates and postgraduates since residents in neurology are those most likely to be in charge of stroke coding. Third, training and reinforcing awareness of medical and non-medical coders for stroke diagnoses could improve the quality of coding, especially if they are permanent staff involved in coding in the neurology department. Fourth, it would be interesting to employ professional coders dedicated to the coding of administrative data, as mentioned in health policy to improve quality. Finally, to improve coding without increasing the workload for senior physicians, a random sample of medical charts for patients hospitalized in several medical units with a diagnosis of stroke could be selected from the FHDDB at fixed intervals. These would then be validated by senior physicians of the neurological department with feed-back to physicians responsible for the coding.
